# Supplementary material for: Resolving the full spectrum of human genome variation using Linked-Reads
Source: Genome Res. 2019 Apr;29(4):635–45. doi: 10.1101/gr.234443.118 (PMC6442396; doi:10.1101/gr.234443.118)
Supplement: Supplemental Material [file supp_gr.234443.118_Supplemental_longranger-2.2.zip › longranger-2.2.tar.bz2!/lr2.2-public/lib/stan/cmdstan/stan/doc/api/html/agrad_2fwd_2functions_2gamma__q_8hpp_source.html]

Stan: src/stan/agrad/fwd/functions/gamma\_q.hpp Source File


|  |  |
| --- | --- |
|  | Stan  2.5.0  probability, sampling & optimization |


- Main Page
- Related Pages
- Namespaces
- Classes
- Files

- File List
- File Members

All Classes Namespaces Files Functions Variables Typedefs Enumerations Enumerator Friends Macros Pages

- src
- stan
- agrad
- fwd
- functions

gamma\_q.hpp

Go to the documentation of this file.

1 #ifndef STAN\_\_AGRAD\_\_FWD\_\_FUNCTIONS\_\_GAMMA\_Q\_HPP

2 #define STAN\_\_AGRAD\_\_FWD\_\_FUNCTIONS\_\_GAMMA\_Q\_HPP

3

4 #include <stan/agrad/fwd/fvar.hpp>

5 #include <stan/meta/traits.hpp>

6 #include <stan/math/functions/gamma\_q.hpp>

7

8 namespace stan{

9

10  namespace agrad{

11

12  template <typename T>

13  inline

14  fvar<T>

15  gamma\_q(const fvar<T>& x1, const fvar<T>& x2){

16  using stan::math::gamma\_q;

17  using std::log;

18  using std::exp;

19  using std::pow;

20  using std::fabs;

21  using boost::math::tgamma;

22  using boost::math::digamma;

23

24  T u = gamma\_q(x1.val\_, x2.val\_);

25

26  T S = 0;

27  T s = 1;

28  T l = log(x2.val\_);

29  T g = tgamma(x1.val\_);

30  T dig = digamma(x1.val\_);

31

32  int k = 0;

33  T delta = s / (x1.val\_ \* x1.val\_);

34

35  while (fabs(delta) > 1e-6) {

36  S += delta;

37  ++k;

38  s \*= -x2.val\_ / k;

39  delta = s / ((k + x1.val\_) \* (k + x1.val\_));

40  }

41

42  T der1 = (1.0 - u) \* ( dig - l ) + exp( x1.val\_ \* l ) \* S / g;

43  T der2 = -exp(-x2.val\_) \* pow(x2.val\_, x1.val\_ - 1.0) / g;

44

45  return fvar<T>(u,x1.d\_ \* der1 + x2.d\_ \* der2);

46  }

47

48  template <typename T>

49  inline

50  fvar<T>

51  gamma\_q(const fvar<T>& x1, const double x2){

52  using stan::math::gamma\_q;

53  using std::log;

54  using std::exp;

55  using std::pow;

56  using std::fabs;

57  using boost::math::tgamma;

58  using boost::math::digamma;

59

60  T u = gamma\_q(x1.val\_, x2);

61

62  T S = 0;

63  double s = 1;

64  double l = log(x2);

65  T g = tgamma(x1.val\_);

66  T dig = digamma(x1.val\_);

67

68  int k = 0;

69  T delta = s / (x1.val\_ \* x1.val\_);

70

71  while (fabs(delta) > 1e-6) {

72  S += delta;

73  ++k;

74  s \*= -x2 / k;

75  delta = s / ((k + x1.val\_) \* (k + x1.val\_));

76  }

77

78  T der1 = (1.0 - u) \* ( dig - l ) + exp( x1.val\_ \* l ) \* S / g;

79

80  return fvar<T>(u, x1.d\_ \* der1);

81  }

82

83  template <typename T>

84  inline

85  fvar<T>

86  gamma\_q(const double x1, const fvar<T>& x2){

87  using stan::math::gamma\_q;

88  using std::exp;

89  using std::pow;

90

91  T u = gamma\_q(x1, x2.val\_);

92

93  double g = boost::math::tgamma(x1);

94

95  T der2 = -exp(-x2.val\_) \* pow(x2.val\_, x1 - 1.0) / g;

96

97  return fvar<T>(u, x2.d\_ \* der2);

98  }

99  }

100 }

101 #endif

stan::agrad::fabs

fvar< T > fabs(const fvar< T > &x)

**Definition:** fabs.hpp:18

gamma\_q.hpp

stan::agrad::tgamma

fvar< T > tgamma(const fvar< T > &x)

**Definition:** tgamma.hpp:15

stan::agrad::pow

fvar< T > pow(const fvar< T > &x1, const fvar< T > &x2)

**Definition:** pow.hpp:17

stan::agrad::fvar

**Definition:** fvar.hpp:13

traits.hpp

stan::math::gamma\_q

double gamma\_q(double x, double a)

**Definition:** gamma\_q.hpp:53

stan::agrad::gamma\_q

fvar< T > gamma\_q(const fvar< T > &x1, const fvar< T > &x2)

**Definition:** gamma\_q.hpp:15

fvar.hpp

stan::math::e

double e()

Return the base of the natural logarithm.

**Definition:** constants.hpp:86

stan::agrad::digamma

fvar< T > digamma(const fvar< T > &x)

**Definition:** digamma.hpp:16

stan::agrad::fvar::d\_

T d\_

**Definition:** fvar.hpp:16

stan::agrad::log

fvar< T > log(const fvar< T > &x)

**Definition:** log.hpp:15

stan::agrad::exp

fvar< T > exp(const fvar< T > &x)

**Definition:** exp.hpp:16

stan::agrad::fvar::val\_

T val\_

**Definition:** fvar.hpp:15


---

[ Stan Home Page ]

*© 2011–2014,
Stan Development Team.*
